# Sauropod Necks: Are They Really for Heat Loss?

**DOI:** 10.1371/journal.pone.0077108

**Published:** 2013-10-30

**Authors:** Donald M. Henderson

**Affiliations:** Royal Tyrrell Museum of Palaeontology, Drumheller, Alberta, Canada; Royal Ontario Museum, Canada

## Abstract

Three-dimensional digital models of 16 different sauropods were used to examine the scaling relationship between metabolism and surface areas of the whole body, the neck, and the tail in an attempt to see if the necks could have functioned as radiators for the elimination of excess body heat. The sauropod taxa sample ranged in body mass from a 639 kg juvenile *Camarasaurus* to a 25 t adult *Brachiosaurus*. Metabolism was assumed to be directly proportional to body mass raised to the ¾ power, and estimates of body mass accounted for the presence of lungs and systems of air sacs in the trunk and neck. Surface areas were determined by decomposing the model surfaces into triangles and their areas being computed by vector methods. It was found that total body surface area was almost isometric with body mass, and that it showed negative allometry when plotted against metabolic rate. In contrast, neck area showed positive allometry when plotted against metabolic rate. Tail area show negative allometry with respect to metabolic rate. The many uncertainties about the biology of sauropods, and the variety of environmental conditions that different species experienced during the groups 150 million years of existence, make it difficult to be absolutely certain about the function of the neck as a radiator. However, the functional combination of the allometric increase of neck area, the systems of air sacs in the neck and trunk, the active control of blood flow between the core and surface of the body, changing skin color, and strategic orientation of the neck with respect to wind, make it plausible that the neck could have functioned as a radiator to avoid over-heating.

## Introduction

Metabolic activity results in the production of body heat, and for large animals elimination of excess body heat is an important factor [1]. As the largest land animals known to have existed, sauropods are expected to have been more susceptible to overheating than even the largest extant tropical forms such as elephants [2]. It has been suggested that the exceptionally long necks of sauropods, in addition to being food-gathering adaptations [3], [4], may have also functioned as a way of cooling the body by using the external surface area of the neck as a radiator [3], [5], [6], [7]. An alternative suggestion involving the long necks of sauropods for cooling was that the jugular veins and carotid arteries could mutually exchange heat during the transit of blood from body to head, and thus avoid suffusing the brain with excessively warm blood [8]. The hypothesis that sexual selection was the primary driver of the evolution of long necks in sauropods [16] has been challenged by [4], and it will not be dealt with further in this paper.

Sauropods are not alone amongst extinct animals in being suggested to have had a specialized anatomy to deal with excess body heat. The dorsal ‘sails’ of late Palaeozoic synapsids such as those of *Dimetrodon* and *Edaphosaurus* have been argued to have functioned as radiators [9]–[12]. Stegosaurian dinosaurs, with their prominent, plate-like osteoderms mounted high on their backs, have also inspired speculations about their ability to lose heat via the extensive vascularization of the plates [13], [14]. However, in the case of the synapsids, it has been shown that the sails were more likely to have been for sexual display based on scaling arguments, and that dorsal sails in small species of *Dimetrodon* would have been ineffective as radiators [15].

There is good evidence that extant animals use the skin as a way eliminate excess body heat. A well-documented case is the use of the surface of the ears and other body regions in African elephants as radiators [40]. This study found that these regions were highly vascularized, and when examined with infrared thermography, these patches showed elevated temperatures that would encourage heat flow away from the body. They also found that the frequency of use of these high-heat patches increased with increasing environmental temperature and with the size of the animals being observed. An earlier study [41] defined an index for the ability of mammals to use the skin as a radiator. This study found that the ability to eliminate heat via the skin scaled positively with body mass. Both of these studies are relevant to the study of heat loss in sauropods.

With the availability of three-dimensional digital models of various sauropods [17], it is possible to test the idea that the geometry of the necks of these animals may have some something to do with the elimination of excess body heat. The hypothesis is that the surface area of the necks, if they really are acting as radiators, should show a correlation with a predicted metabolic rate. When plotted on a log-log plot of the type typically used for analyses of scaling relationships [18], the scaling coefficient (the slope of the regression line) for neck area as a function of metabolism should show a slope of at least 1.0. A slope less than unity would imply that the neck area was not keeping pace with increasing metabolic heat production as body size increased during the evolution of increasingly larger and larger sauropods, and unlikely to be correlated with heat loss.

## Materials and Methods

### Materials

**Figure 1:**
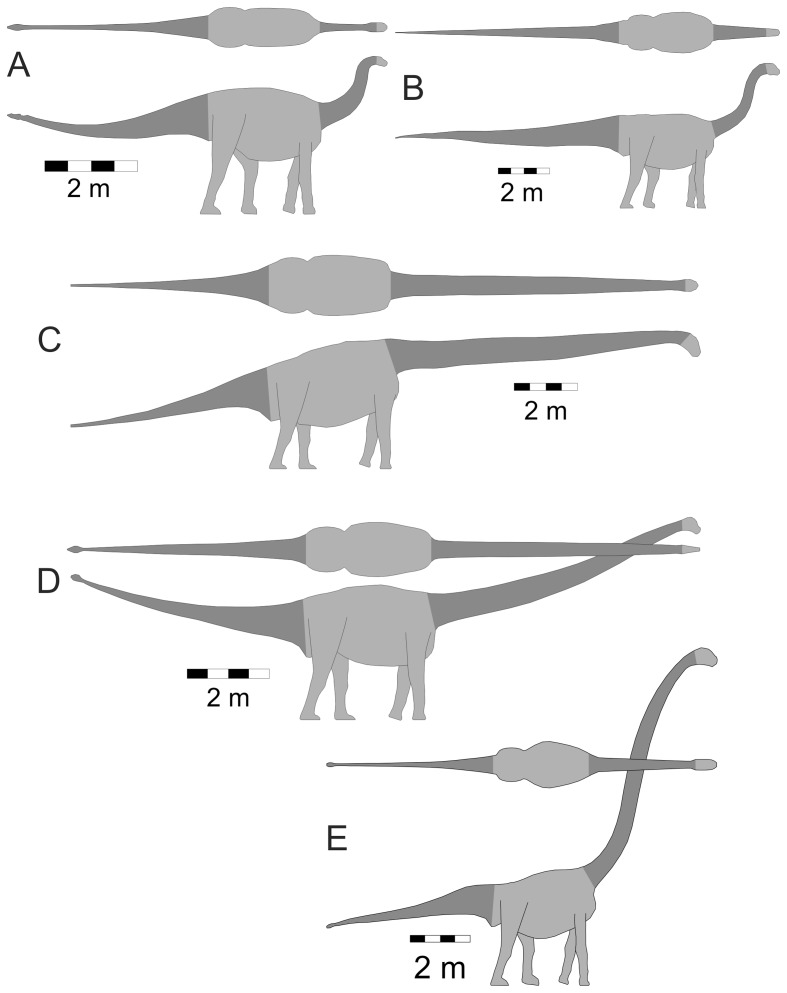
Basal sauropodomorphs. Isometric dorsal and lateral views of the taxa used in the present study. The extent of the neck analyzed for surface area is highlighted with the dark grey colour on each model view. (A) *Shunosaurus lii*. (B) *Patagosaurus fariasi*. (C) *Mamenchisaurus hochuanensis*. (D) *Mamenchisaurus youngi*. (E) *Omeisaurus junghsiensis*. See Table 1 for sources used to generate the models.

**Figure 2:**
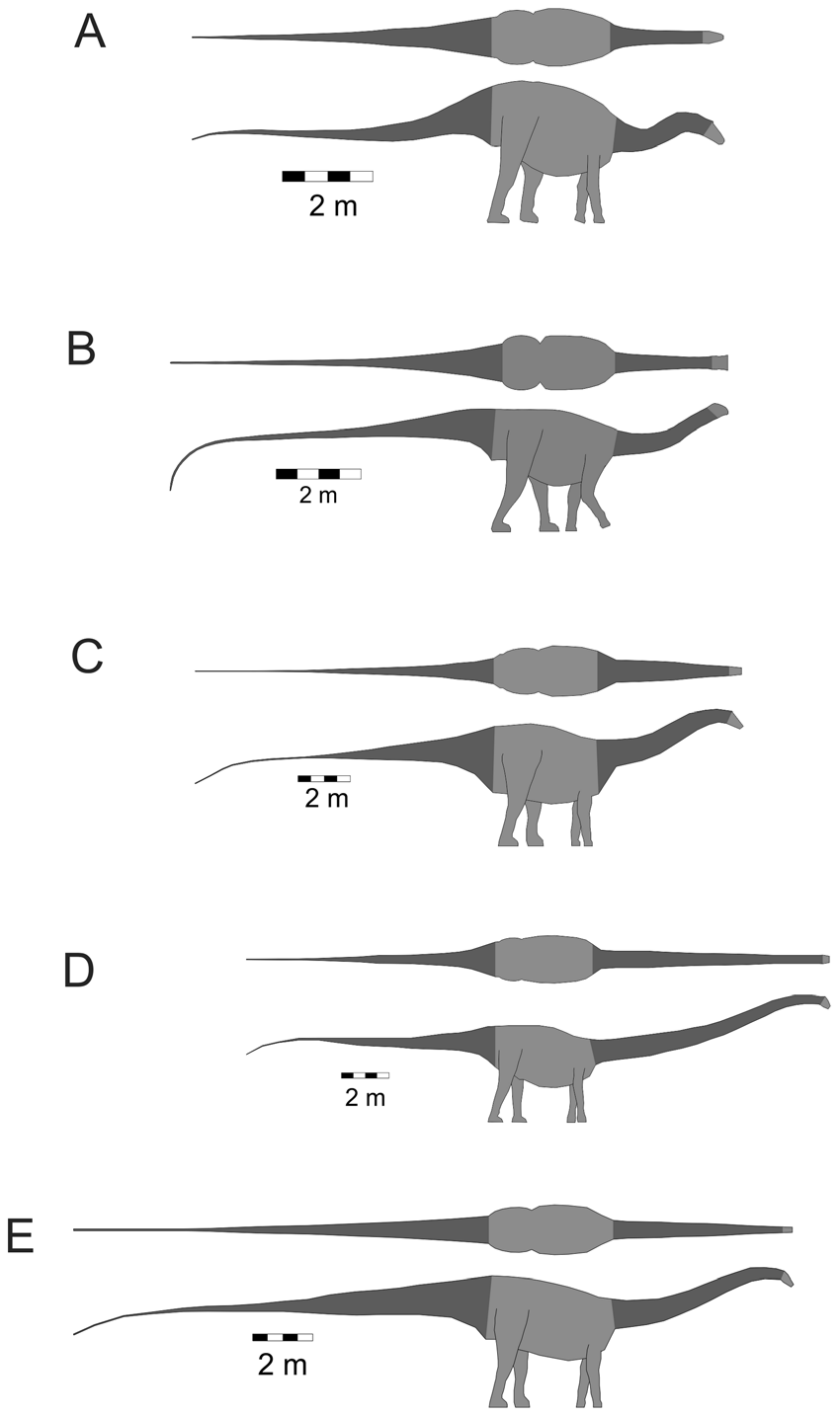
Diplodocoid sauropodomorphs. Isometric dorsal and lateral views of the taxa used in the present study. (A) *Dicraeosaurus hansemanni*. (B) *Nigersaurus taqueti*. (C) *Apatosaurus louisae*. (D) *Barosaurus lentus*. (E) *Diplodocus carnegii*. Details as per Figure 1.

**Figure 3:**
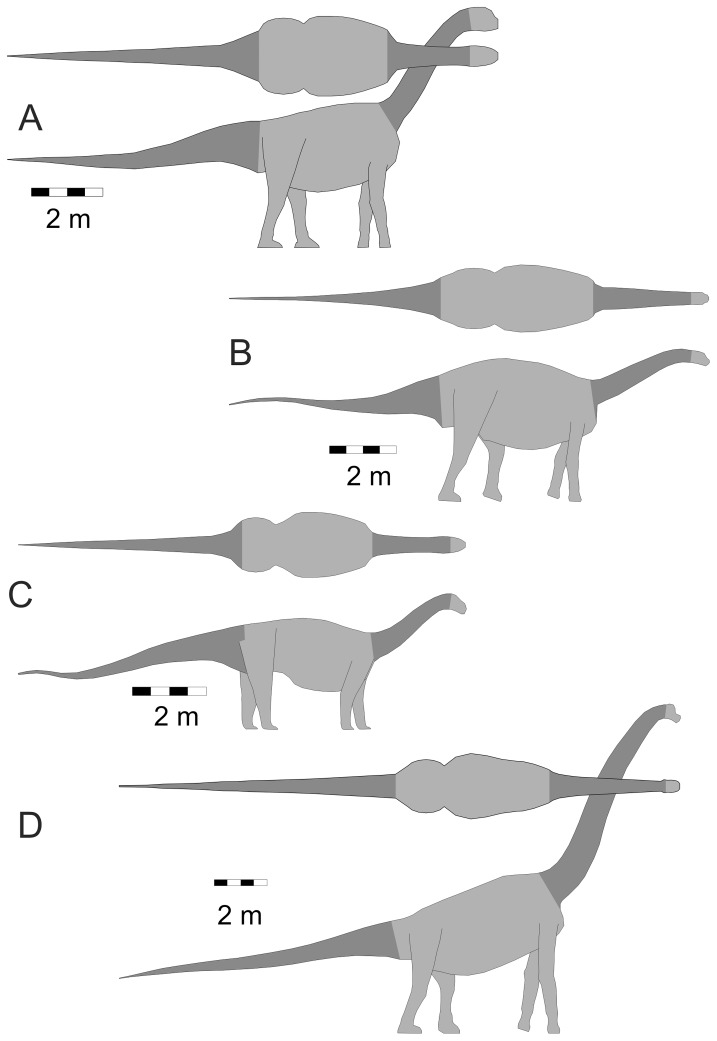
Macronarian sauropodomorphs. Isometric dorsal and lateral views of the taxa used in the present study. *Jobaria tiguidensis* is not shown for space reasons. (A) *Camarasaurus lentus*. (B) *Haplocanthosaurus priscus*. (C) *Saltasaurus loricatus*. (D) *Brachiosaurus brancai*. Details as per Figure 1.

Three-dimensional digital models of sixteen sauropods were generated using published life restorations that showed the animals in lateral and dorsal views. The taxa modeled and their component masses, body lengths and image sources are listed in Table 1. The shapes of the models were obtained using the three-dimensional slicing method outlined in [19]. As the extent of the neck was the body region of primary interest, it was consistently delineated in all the models. The posterior end of the neck was set at a slice crossing the axial body tangent to the anterior-most point of the illustrated pectoral girdle. The anterior neck limit was set at a slice tangent to the posterior-most point of either the cranium or the mandible, whichever was the most posterior. The extent of the neck on all the models is shown with the dark shading in the cervical region in figures 1, 2 and 3. As a comparative check on the area of the neck relative to the total body surface area, the surface area of the tail was also computed. The posterior limit for a tail was its distal-most tip, while the anterior limit was the slice that was tangent to the most posterior component of the pelvis, either the ischium or the ilium, depending on the species. The extent of the tail is also highlighted in the model figures with a dark shading.

**Table 1:** Length, masses and sources for sauropod body models.

	Body Length (m)	Total Body Mass (t)	Axial Mass (t)	Single Leg Mass (kg)	Single Arm Mass (kg)	Image Source	Figure Abbreviation
*Apatosaurus louisae*	21.8	16.4	13.2	1.27×10^3^	302	[30]	A.l
*Barosaurus lentus*	25.0	15.8	13.7	817	233	[30]	B.l
*Brachiosaurus branchai* *	25.8	26.3	21.1	1.63×10^3^	945	[29]	B.b
*Camarasaurus lentus* (adult)	15.5	12.3	10.3	724	257	[30]	C.l(a)
*Camarasaurus lentus* (juvenile)	5.73	0.639	0.560	28.6	11.1	[30]	C.l(j)
*Dicraeosaurus hansemanni*	12.1	4.35	3.73	258	48.0	[30]	D.h
*Diplodocus carnegii*	24.9	11.9	9.97	829	159	[30]	D.c
*Haplocanthosaurus priscus*	15.0	13.5	11.4	905	166	[30]	H.p
*Jobaria tiguidensis*	18.2	22.4	18.7	1.40×10^3^	449	[24]	J.t
*Mamenchisaurus hochuanensis*	20.6	12.8	11.4	453	228	[30]	M.h
*Mamenchisaurus youngi*	16.1	5.36	4.36	335	169	[30]	M.y
*Nigersaurus taqueti*	14.1	3.64	3.09	326	105	[39]	N.t
*Omeisaurus junghsiensis*	18.3	6.73	5.73	364	138	[30]	O.j
*Patagosaurus fariasi*	16.5	7.88	6.89	344	150	[30]	P.f
*Saltasaurus loricatus*	12.8	6.87	5.63	438	182	[30]	S.l
*Shunosaurus lii*	9.02	2.16	1.68	173	67.0	[30]	S.li

* Tail extended relative to published illustration based on other sauropod tail and body proportions [17].

### Methods

#### Body Mass Estimations

Determination of the body masses of the sauropod models requires estimating the volumes of the various body parts and assigning those parts specific density values. Volumes of the axial body and the limbs of the sauropod models were calculated using the three-dimensional mathematical slicing method of [19]. The volumes were then multiplied by particular density values to compute their masses. The selection of density values to use were based on observations of living animals with the limbs and tails being assigned a basic density equal to that of water – 1,000 gm/l. The combination of the evidence for extensive pneumatization of the precaudal sauropod axial skeleton [7], [20], the suggestion that sauropods would have required a respiratory system similar to that of birds [21], and the observation that the air sacs of modern birds occupy about 15% of the trunk volume [22], led to the pelvic and trunk regions having their basic density of 1000 gm/l reduced by 15% to 850 gm/l. The pneumatized neck of a goose was observed to have a density of 300 gm/l [23], and this value was used for the necks and heads of all the models. An additional form of mass reduction was done with the inclusion of a lung cavity within the chest region. Lacking any other objective way of estimating a lung volume for sauropods, the scaling relationship between body mass and lung volume determined for birds [18] was used. See [17], [25] for more details on assigning densities to pneumatized bodies.

#### Metabolic Rate

The main purpose of accurately determining the body masses of the various sauropods was to provide a base for estimating metabolic rate. Following the general rule that basal metabolic rates scales to body mass raised to the ¾ power [18], a provisional metabolic rate, assuming a unitary coefficient, was computed for all the models. The coefficient of 1.0 was chosen in light of the controversy about the metabolic rates of dinosaurs in general, and sauropods in particular [6], and the probability that it decreased during ontogeny [26], [27]. For the purposes of the present study it is not the actual metabolic rate that is of interest, but how the metabolic exponent, 0.75, compares with the exponent associated with body surface magnitude.

It must be noted that there is mounting evidence that the ¾ scaling factor cannot be arbitrarily applied to all animals. Not only might it differ from ¾, but that it depends on whether the animal is an ectotherm or an endotherm [42]. For the present study it will be assumed that the sauropds were functional endotherms due to their large body size, and based on the analysis in [42], their metabolic scaling factor would not have exceeded 0.75. See Discussion for the implications of different metabolic scaling factors for the present study.

#### Surface Area Calculations

**Figure 4:**
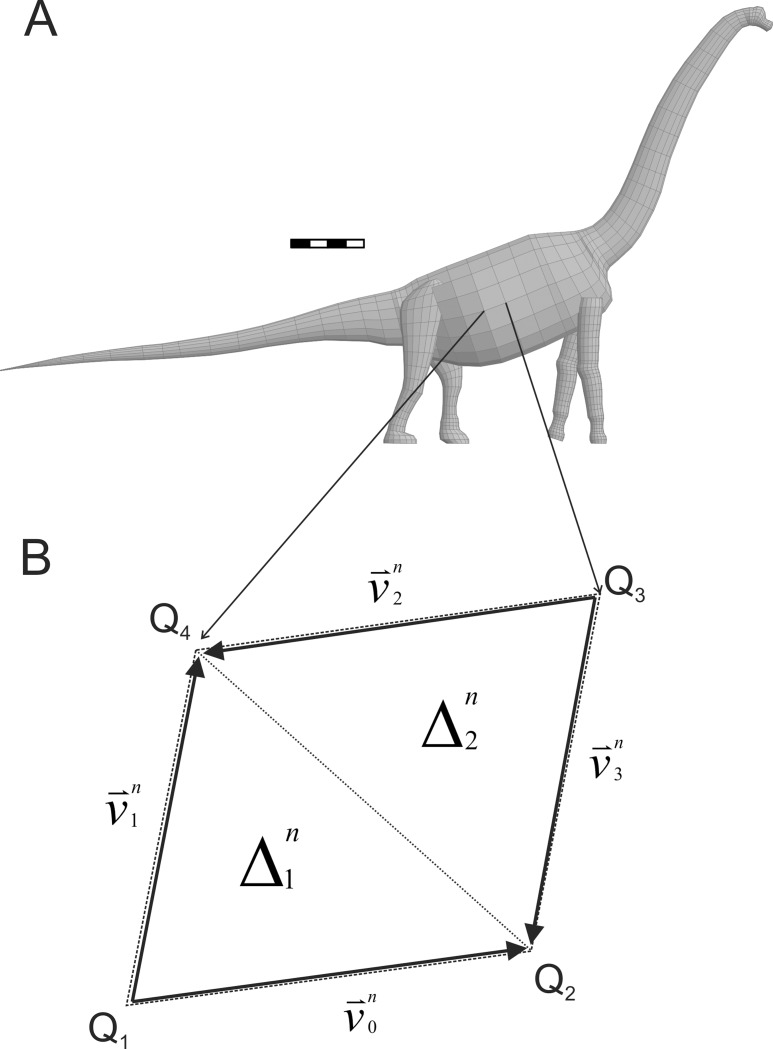
Determining external surface area. (A) *Brachiosaurus* model showing the quadrilateral tessellation used to compute the surface area of the model. The scale bar is 2 m. (B) Decomposition of the n^th^ body surface quadrilateral into two sub-triangles 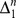 and 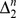. Q_1_, Q_2_, Q_3_ and Q_4_ are the four points defining the vertices of the quadrilateral. The edges of the triangles are represented by vectors between the vertices. One half of the magnitude of the vector cross-product of a pair of co-terminal vectors, eg. 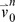 and 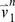, gives the area of the triangle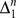. See Methods: Surface Area Calculations for more detail.

The forms of the digital sauropod models make it relatively easy to compute their surface areas. The three-dimensional mathematical slicing method used to define a body shape uses ellipses to represent the slices which are transverse sections of the limbs and axial body. These ellipses are further decomposed into discrete sets of points with the same number of points used for all the slices used to define a particular body region. The homologous points on adjacent slices can be linked together and this results in the surface of the body region being decomposed into a set of quadrilateral facets. The full quadrilaterally tessellated surface of the model of *Brachiosaurus* is shown in figure 4A.

The total external area of a limb or axial body is determined by summing the areas of all the component polygons. To compute the area of a polygon each one is divided diagonally into two triangles, and the two sides of each triangle are represented by vectors. (figure 4B). For the n^th^ polygon the vector pair 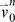 and 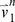 define two sides of the first triangle, while 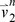 and 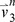 define two sides of the second triangle, and the expression to compute the total surface area of a model component, 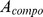, is:
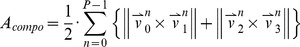
(1)where 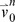, 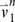, 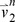 and 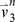 are the vectors defined between, respectively, the first and second, first and fourth, third and fourth, and third and second perimeter points on the n^th^ polygon, and *P* is the number of polygons comprising the model component. This expression computes the vector cross-products of the respective vector pairs defining the two triangles to get the areas of the parallelograms spanned by the vector pairs, determines and sums the magnitudes of the two areas, and then divides this result by two as we only need the area of the triangles, not the full parallelograms. Using the two planar triangles to approximate the surface area of the curved quadrilateral surface results in an average underestimate of approximately 0.6%.

## Results

**Figure 5:**
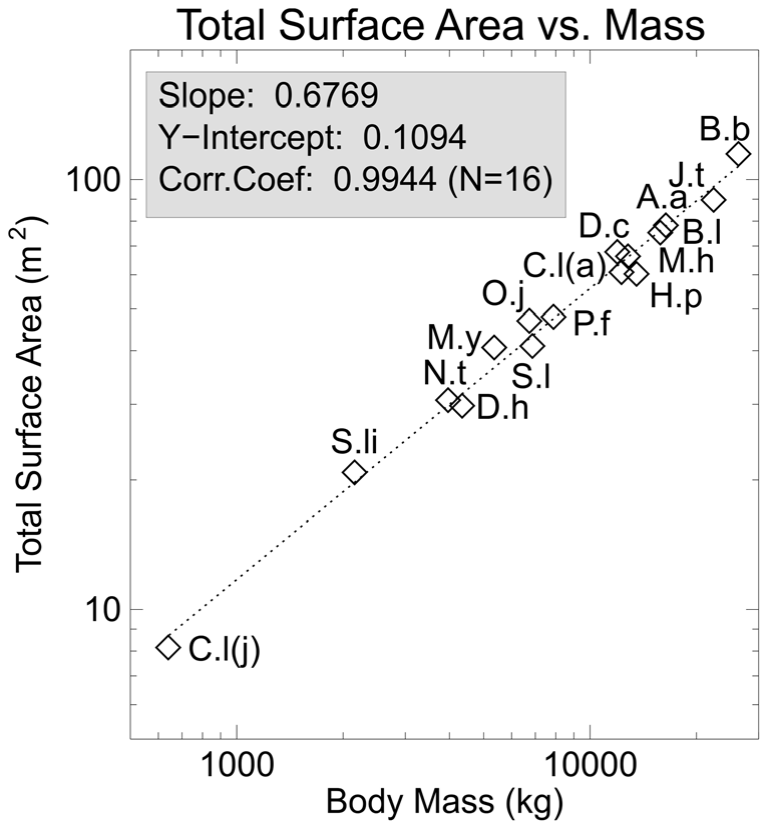
Surface area versus body mass. Log-log plot of total external body surface area plotted against total body mass for all the sauropod models. Note that the slope of the fitted regression line is close to that predicted for isometric size increase – 0.67. See Table 1 for taxa abbreviations.

The computed total and regional surface areas for all the models are summarized in Table 2. Figure 5 shows total surface area plotted against body mass with a slope of 0.6769 and a very strong correlation coefficient of 0.9944. Surprisingly, the slope of the regression is very close to that expected if the animals increased their size isometrically where the expected slope would be 2/3 (0.66666…) [18]. The fact that the slope is greater than 2/3 indicates that surface area does increase slightly faster than body mass.

**Table 2:** Sauropod model surface areas. All areas measured in square metres.

	Total	Axial Body	Single Leg	Single Arm	Neck	Tail
*Apatosaurus louisae*	78.2	53.8	9.04	3.15	16.5	4.75
*Barosaurus lentus*	75.2	56.4	6.57	2.83	19.5	3.94
*Brachiosaurus branchai*	114	79.2	10.7	7.12	21.5	16.5
*Camarasaurus lentus* (adult)	60.8	42.2	6.36	2.97	9.49	11.8
*Camarasaurus lentus* (juvenile)	8.15	5.65	0.860	0.389	1.03	1.56
*Dicraeosaurus hansemanni*	29.8	20.9	3.39	1.03	3.33	6.92
*Diplodocus carnegii*	67.8	50.6	6.55	2.06	9.96	19.3
*Haplocanthosaurus priscus*	60.3	40.5	7.51	2.39	7.28	8.68
*Jobaria tiguidensis*	89.7	60.5	9.99	4.57	14.5	14.2
*Mamenchisaurus hochuanensis*	66.2	51.2	4.63	2.88	18.9	10.0
*Mamenchisaurus youngi*	40.7	28.4	3.79	2.36	8.94	6.88
*Nigersaurus taqueti*	30.6	19.9	3.66	1.69	3.23	6.51
*Omeisaurus junghsiensis*	46.9	33.9	4.34	2.19	12.3	6.69
*Patagosaurus fariasi*	47.9	34.8	4.36	2.22	5.28	12.6
*Saltasaurus loricatus*	41.0	27.7	4.32	2.37	4.45	8.23
*Shunosaurus lii*	20.8	13.1	2.62	1.23	1.67	4.17

**Figure 6:**
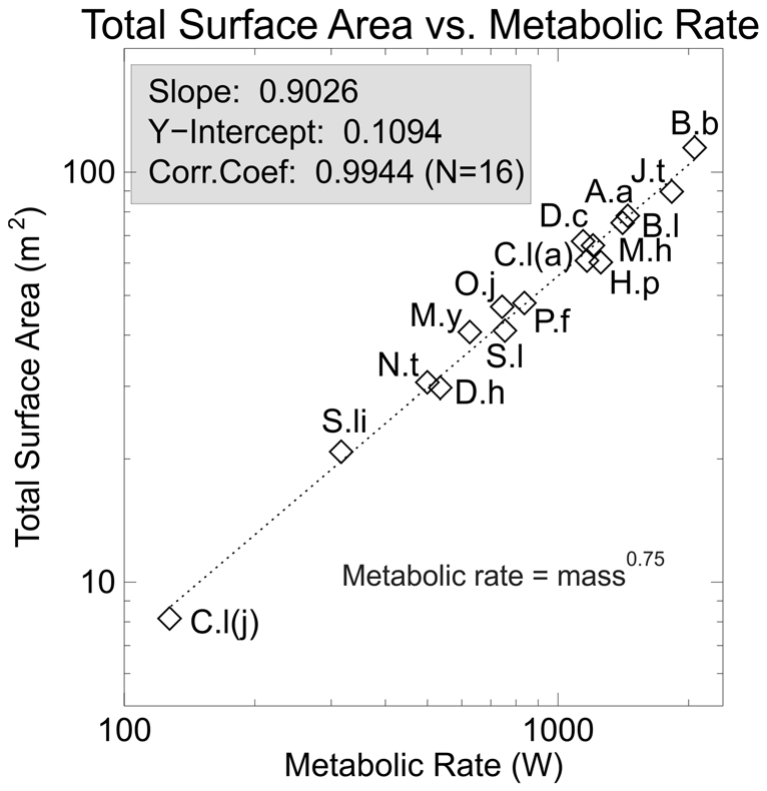
Surface area versus metabolic rate. Log-log plot of total external body surface area plotted against a dimensionless metabolic rate proportional to body mass raised to the ¾ power. Note that the fitted regression line is less than 1.0 implying that surface area will lag behind metabolic heat production with phylogenetic size increase. This could lead to overheating due to reduced relative surface area available to radiate excess heat.

Figure 6 shows total surface area plotted against the assumed metabolic rate. The slope can now be seen to be greater than that predicted from isometry, but is still less than 1.0, implying that body heat production might exceed the ability of the body to eliminate it, leading to overheating. However, this ignores internal blood circulation and the ability of animals to control the flow of heat from the body core to the surface [6].

**Figure 7:**
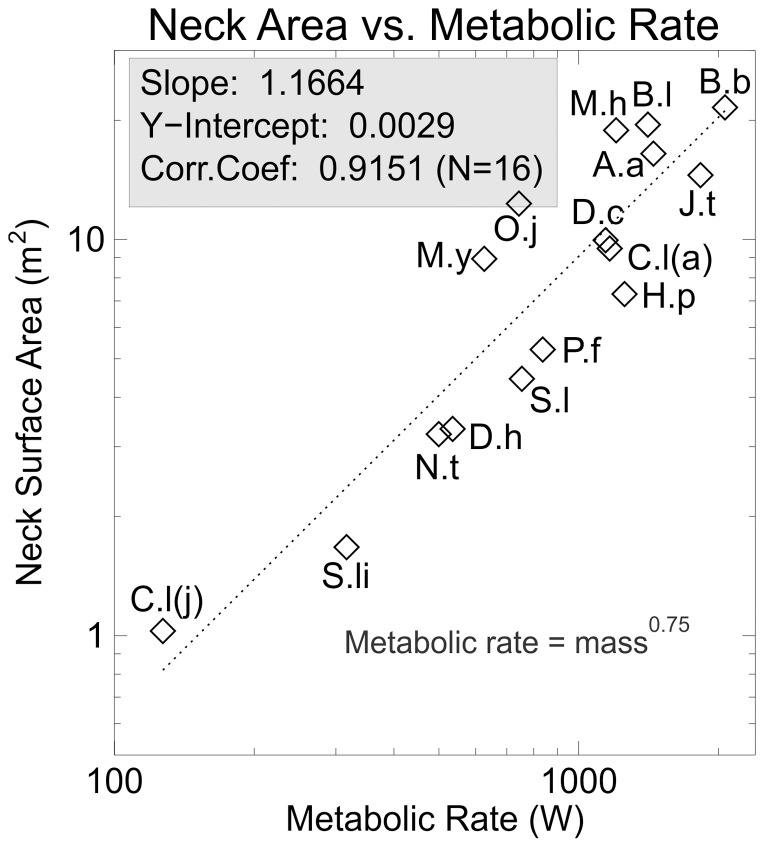
Neck area versus metabolic rate. Log-log plot of neck surface area versus a dimensionless metabolic rate. The scaling exponent is greater than 1.0 implying that neck area increases faster than expected if the animals increased neck size isometrically. The increased neck area could function as a radiator to eliminate excess body heat.

Figure 7 shows neck surface area plotted against metabolic rate. The computed slope, 1.1664, is now greater than 1.0. It is clear that while the increase in overall body surface area lags behind that of metabolic rate, the neck area shows a positively allometric size increase. This suggests that the neck may have a special role in the elimination of body heat. Those sauropods with noticeable short necks relative to body size – *Shunosaurus*, *Nigersaurus*, *Dicraeosaurus*, *Saltasaurus*, *Patagosaurus*, and *Haplocanthosaurus* do lie below the computed regression line, but closely parallel it. This indicates that a similar scaling factor applies to these taxa, but with a smaller Y-intercept.

**Figure 8:**
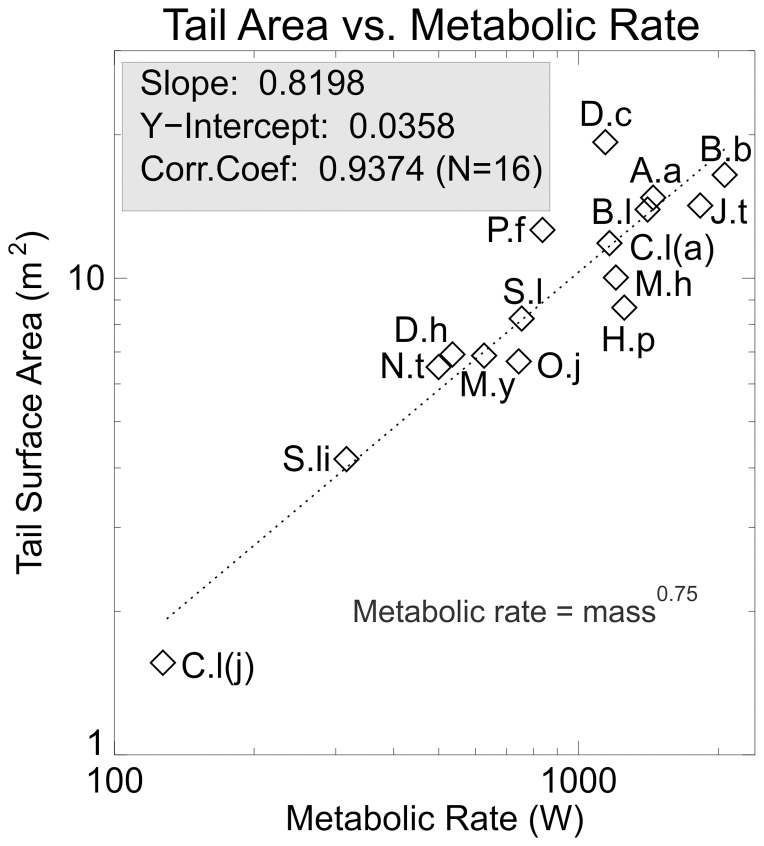
Tail area versus metabolic rate. Log-log plot of tail surface area versus a dimensionless metabolic rate. The scaling exponent is less than 1.0 implying that the tail alone would not be effective in dumping excess body heat.

The other elongate structure projecting from the body of a sauropod is the tail, and it was felt that a comparison of tail surface area scaling with that of the neck might be informative. Figure 8 shows the surface area of the tail plotted against metabolic rate. The slope of the regression, 0.8198, is less than that for both the neck and the body as a whole. This would seem to indicate that the tail is either not specially adapted for eliminating excess body heat, or that its role in heat loss was a passive one.

## Discussion

The present study lumps together 14 genera of which only six were sympatric, the others existed at different times and on different continents. This lumping combines animals that would have experienced different climates with different diurnal and seasonal temperature changes, possibly obscuring any anatomically significant patterns related to the dissipation of excess body heat. The sympatric six are those specimens from the Late Jurassic Morrison Formation. *Apatosaurus*, *Camarasaurus* and *Diplodocus* are commonly found in close temporal association [28]. Less frequently, these three genera are variably associated with *Barosaurus*, *Brachiosaurus* and *Haplocanthrosaurus* at different levels in the Morrison Formation [28]. However, in an attempt to explain the peculiar anatomy of sauropods as it relates to their control of body temperature, it was felt that it was best to combine all the taxa to have as large a sample size as possible. The results and inferences derived from this study must be taken as preliminary as new discoveries will add to our knowledge of the group.

The evidence that there is no one single metabolic scaling factor of 0.75 for all animals, and that it was most likely less than 0.75 for endotherms [42], increases the contrast between it and the neck area scaling factor of 1.17 identified for sauropods. This probable increased contrast implies an even greater ability in these animals to eliminate excess body heat via the neck. The findings that metabolic scaling varies between different groups of terrestrial vertebrates [43], suggests that different types of sauropods, living at different times and places all over the world, may have had different metabolic scaling factors. This sort of unknown, but likely variability, means that the findings of this study must be seen as preliminary. However, the existence of variable metabolic scaling does not invalidate the basic finding of the present study.

It has been suggested that the overall attenuated body shape of sauropods with a long thin tail and neck was a sign that they were trying to maximize surface area to avoid overheating. From the present analysis it would seem to not be the case. The finding that surface area scales almost isometrically with body mass in the present sample of sauropods (Fig. 5) may be a true biological signal, or it may be an artifact of the restorations. Most of the illustrations used to generate the models were done by [29], [30] (Table 1). Given that most sauropods are known from incomplete material [31], there may be a stylistic influence that results in isometric restorations. However, the morphological conservatism of the basic sauropod body plan [31], could be used as an argument that the near-isometric scaling observed is genuine. It has been observed that the expression for surface area (SA) as a function of body mass (M) in vertebrates, SA = kM^0.67^, the value of ‘k’ typically ranges between 9 and 11 [32], with 10 being an acceptable compromise when mass is measured in kilograms and area measured in decimeters [18]. Converting the square meters used in the present study to decimeters means changing the ‘k’ value (“Y-Intercept” of Fig. 5) from 0.1094 to 10.94, a value similar to that seen in much smaller extant vertebrates.

**Figure 9:**
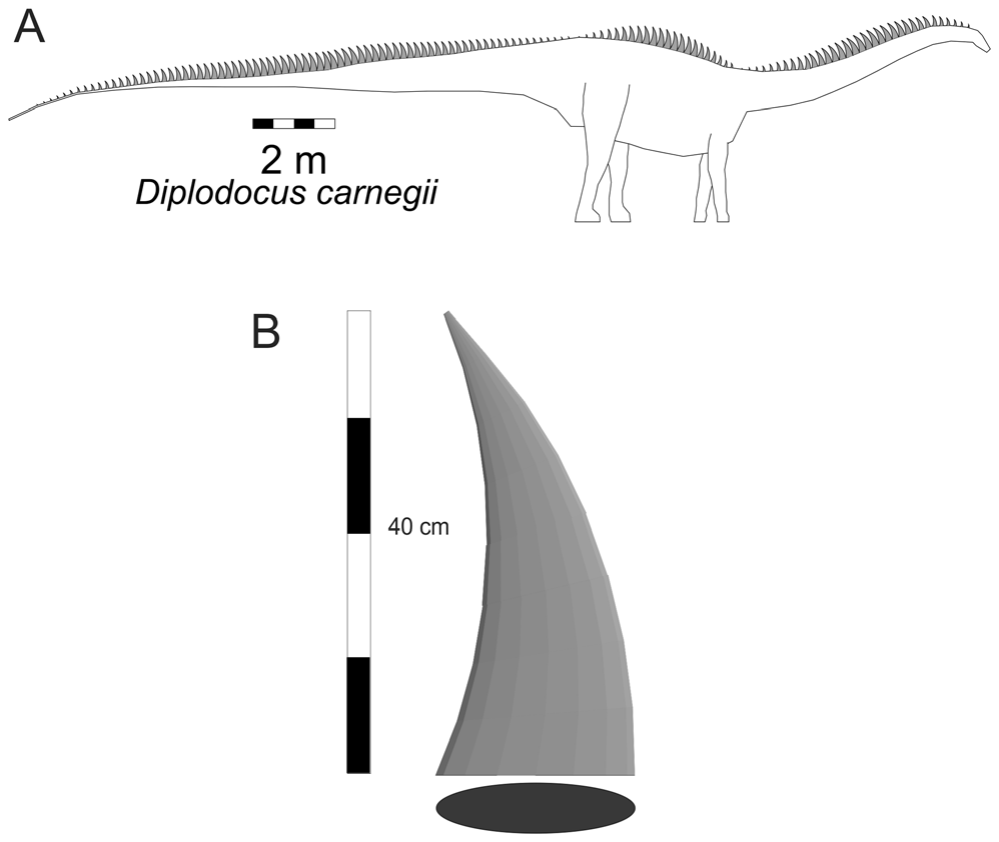
Neck length versus body mass. Log-log plot of absolute neck length plotted against body mass. The great deal of scatter, as indicated by the low correlation coefficient, highlights the variety of neck lengths exhibited by sauropods, and makes it difficult to make broad generalizations about neck function in these animals.

With the discovery of dermal spines running along the dorsal midline of the body of *Diplodocus* [44], there is the possibility that the dermal spines themselves could have contributed to increasing the surface area of the animal and further enhancing heat loss. As a test of this idea, a diplodocid model with a full set of dermal spines was generated using the configuration shown in [44]. The tallest of these spines was set to 40 cm, and the heights of the others were set as a sine function of the position of the dermal spine along one of the three body segments – tail, trunk, neck – with the tallest always being in the middle of the body segment. (Fig. 9A). Representing this 40 cm tall spine as a three-dimensional mesh with a narrow elliptical cross-section (Fig. 9B), its total lateral surface area is 855 cm. For the sets of dermal spines along the tail, trunk and neck body segments their combined areas are 4.55 m^2^, 1.38 m^2^ and 2.20 m^2^, respectively, and the total spine area is 7.94 m^2^. The total surface area of the axial body and limbs of the *Diplodocus* model is 67.8 m^2^, so the full dermal spine area represents 11.7% of the body area. If these spines were vascularized then they would have a significant potential to act as radiators. However, there is a great deal of uncertainty about the size, internal structure, and distribution of these spines on the body. Until better fossil material becomes available, the importance of any dermal spines for heat loss in sauropods will have to remain speculative.

**Figure 10:**
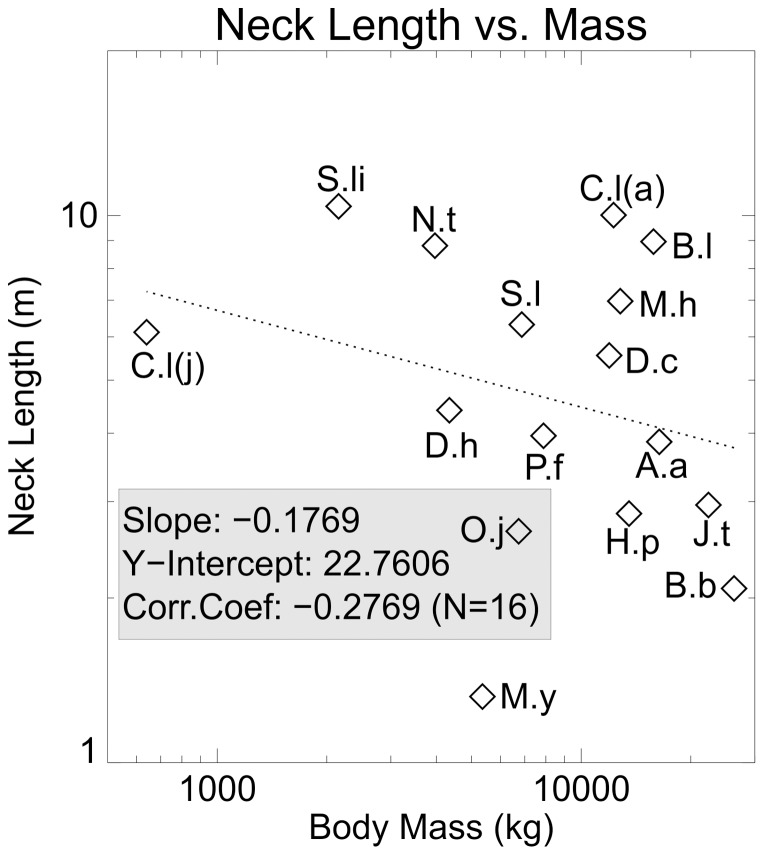
*Diplodocus* with dermal spines. Alternate model with sets of dermal spines running along the dorsal midline of the body. The tallest spine in each of the tail, trunk and neck is 40[44].

It appears to be generally accepted that the long necks of sauropods were food gathering organs that allowed the animal to stand in one place, thus minimizing the energetic cost of movement, while exploiting the large volume of foliage that would be within reach [4], [33]. A larger animal needs more food, so it might be expected that a larger animal would have a longer neck. Figure 10 plots neck length against body mass. As can be seen, there is a weak trend for neck length to decrease relative to body mass. However, there is a great deal of scatter in the data as indicated by the low correlation coefficient. Absolute neck length does not follow the same trend as neck area. This scattered plot appears to be a result of lumping diverse group of animals together.

Although morphologically conservative, sauropods were a diverse group of dinosaurs with approximately 200 named species whose remains are found on all continents, and inhabited a variety of environments [34]. As well as having a wide geographic extent, the group also existed for a long time, from the Late Triassic to the end of the Cretaceous, a period of approximately 150 million years. The combination of long duration and wide dispersal means that these animals must have experienced a diversity of vegetation types that could be exploited as a food source [35]. As can be seen from the few complete skulls known for these animals, they had diverse skull shapes and dentitions [36], with the inference that there was diversity not only in body size, but also in feeding styles and foods eaten. The poor correlation of neck length with body mass might be a reflection of the diversity of feeding strategies used by sauropods. Neck length is intimately associated with the total area of the neck. This diversity of neck lengths makes it difficult to summarize the contribution of the neck to possible cooling strategies used by sauropods.

The thermal environment of any animal is complex with various sources and sinks of heat [6], [37], and these will affect how an animal is able to control its body temperature. For elimination of body heat there is evaporative, convective, conductive, and radiative cooling. For sauropods living in semi-arid landscapes such as that recorded by the Morrison Formation [28], water conservation may have been a key concern, thus limiting the potential for evaporative cooling. At large body size the ability of animals to use convective means to eliminate excess heat is reduced due to the thickening of the thermal boundary layer around the body with increasing size [6]. The limited degree of contact with the ground through their feet would have made conductive cooling unlikely in sauropods. However, the capacity for radiative heat loss does not diminish with large body size [6], and the relative increase in neck area with increasing size suggests that sauropods could have been using this method to avoid overheating. Additionally, it has been shown that heat exchangers such as the long limbs of sauropods, or the frills of ceratopsians, are much more effective at large body size [6]. These authors also suggested that the necks of sauropods could be effective radiators. The effectiveness of necks as a radiator will depend on the external temperature and infrared thermal radiation from the environment in which their sauropod owners inhabit [6]. These aspects of the physical environment for extinct sauropods are almost impossible to quantify, and they would not be the same for all sauropods as they lived in different climates at different times in Earth history. Again, this uncertainty makes it difficult to claim with absolute confidence that the necks functioned as cooling structures, but does not render the idea implausible.

The above discussion has tacitly assumed that heat loss would be passive, with conduction from the core of the body to the external surface being the only mechanism. However, it is well known that animals such as crocodilians are able to increase and decrease the flow of blood between the core and surface of the body to affect heating and cooling rates [14], and birds are able to increase breathing rates to effect elimination of excess body heat (“panting”) [38]. There is also the ability of lizards to change their skin color from light to dark to improve their ability to absorb the warmth of the sun, with the converse being that a lighter colored skin will absorb less infrared radiation and slow the rate of heating. The cervical vertebrae of most sauropods show deep pleurocoels on the sides of their centra, and well- developed systems of airways within the centra (pneumatization), and it is hypothesized that sauropods could have used physiological mechanisms similar to those of birds to facilitate cooling of the body [7]. The scaling exponent for the relationship between whole body surface area and metabolic rate for the sauropod sample is less than 1.0. However, neck area alone has a scaling exponent greater than 1.0. This could be interpreted as showing that the neck is a specialized organ for the elimination of body heat in sauropods. The combined effects of increased neck surface area for radiative heat loss; the elaborate system of air sacs intimately associated with the core of the body; a second system of air sacs in the neck in close association with the blood vascular system of the neck, and close to the external neck surface; and the possibility of changes in neck skin color could all contribute to making the neck a highly effective structure for the elimination of excess body heat. Lastly, modeling studies [6] have shown that increased wind speeds would have a significant cooling effect on large dinosaurs. Properly orienting the sauropod neck with respect to the wind would maximize the ability of the moving air to reduce the thermal boundary layer and further improve heat loss.

## Conclusions

The suggestion that the necks of sauropods functioned as cooling structures has not been rejected by the present study. A positive allometric trend with a scaling exponent of 1.1664 for neck surface area to increase with increasing metabolic rate is considered to be an important indicator that the neck was capable of being a radiator. In combination with a system of thoracic and cervical air sacs, and active control of blood flow from the internal regions of the body to the surface, the neck would have made an effective heat loss structure. The effectiveness of a cooling mechanisms such as convection would be reduced for sauropods on account of their immense size. Similarly, evaporative cooling would seem unlikely given their apparent preference for dry habitats. Their unique anatomy, extreme body size, and lack of living descendants make sauropods difficult to interpret as living organisms. However, observations of analogous structures and cooling functions in living relatives such as birds and crocodiles enables plausible interpretations of aspects of sauropod thermal biology.
